# Handling by avian frugivores affects diaspore secondary removal

**DOI:** 10.1371/journal.pone.0202435

**Published:** 2018-08-29

**Authors:** Tadeu J. Guerra, João V. S. Messeder, André J. Arruda, Lisieux F. Fuzessy, Roberta L. C. Dayrell, Frederico S. Neves, Fernando A. O. Silveira

**Affiliations:** 1 Departamento de Botânica, Universidade Federal de Minas Gerais—Belo Horizonte, Brazil; 2 Departamento de Biologia Geral, Universidade Federal de Minas Gerais—Belo Horizonte, Brazil; Indiana University Bloomington, UNITED STATES

## Abstract

The balance between the costs and benefits of fleshy fruit production depends on the feeding behavior of their seed dispersers, which might effectively disperse seeds to farther areas or drop beneath parent plants some diaspores they handle during frugivory bouts. Nevertheless, the consequences of variation in fruit handling by primary seed dispersers on the secondary removal of diaspores remains poorly understood. We conducted a field study to determine how variation in fruit handling by avian frugivores affects short-term secondary removal of *Miconia irwinii* (Melastomataceae) diaspores by the ground-dwelling fauna in *campo rupestre* vegetation, southeastern Brazil. We conducted factorial experiments manipulating: (1) different outcomes of primary fruit/seed removal by birds, (2) distances of diaspore deposition from conspecifics, and (3) the access of ants and vertebrates to diaspores. We showed that secondary removal of diaspores was highly variable at the population scale, with an overall low removal rate by the ground-dwelling fauna (13% seeds, 19% fruits). However, we found that gut-passed seeds embedded in bird feces were less removed than seeds expelled from fruits. Gut-passed seeds were more likely to be removed by ant species acting as secondary dispersers, whereas pulp-free seeds dropped by birds were likely to interact with potential seed predators, including ants and rodents. We found no clear effect of dispersal from parent plant vicinity on seed removal, but fruit removal was significantly higher near parent plants. Partially defleshed fruits were more removed than intact fruits. The removal of fruits by ant and vertebrate rescuers, including lizards and birds, might reduce the costs of interactions with less effective dispersers that drop partially defleshed fruits under parent plants. Our study highlights that variation in fruit handling by primary avian seed dispersers mediate subsequent interactions among discarded diaspores and ground-dwelling animals, potentially affecting final seed fates. Moreover, we argue that escape-related benefits of dispersal can be contingent on how primary dispersers handle and discard seeds.

## Introduction

One of the main benefits of seed dispersal relates to escape from density-dependent attack by pathogens and predators near parent plants [1–3]. Endozoochory, in particular, involves fruit consumption and seed passage through vertebrate guts, which also may benefit plants by enhancing seed germination through coat scarification and release from germination inhibitors [4,5]. Frugivores benefit plants when they act as legitimate seed dispersers, those able to ingest seeds embedded within the fruit pulp, to defecate or regurgitate viable seeds in safe sites far from parent plants [3]. However, plants also have to cope with the costs of interacting with seed predators or with frugivores that consume the fruit pulp while dropping the diaspores at the proximity of parent trees, thus acting as ineffective seed dispersers [6,7]. Therefore, interactions with multiple frugivore species varying in their effectiveness as seed dispersers encompass both costs and benefits to fruiting plants.

The fate of seeds dispersed by frugivores also depends on their secondary displacements after reaching the soil [8]. Seed predators or secondary dispersers may rearrange the spatial distribution of diaspores handled and discarded by primary dispersers, thereby directly affecting their fate [9–11]. For instance, birds are the primary seed dispersers of a large number of plant species in Neotropical ecosystems [12] and ants frequently interact with fallen diaspores of ornithochoric plants across different vegetation types [13–17]. Ants might remove seeds defecated in sites distant from the parent plants [13,15] or even rescue diaspores dropped beneath parent plants by avian frugivores [18,19]. Consequently, ground-dwelling animals interacting with diaspores can thus shift the fate of seeds dropped beneath parent plants by ineffective dispersers as well as of seeds effectively dispersed to farther areas by frugivores.

The way primary dispersers handle and discard seeds might affect the subsequent interactions with seed predators and secondary dispersers [20,21]. Passage through the gut of avian dispersers can change the chemical condition of chili pepper seeds reducing volatile attractants to ant seed predators and pathogen attack [22,23]. Indeed, bird-pecked fruits with pulps partially defleshed and seeds embedded in the feces of avian frugivores can attract more ant species than seeds enclosed within intact fruits [24]. Moreover, rodent predators may remove most of the seeds regurgitated by avian dispersers, whereas seeds present in bird feces can be removed by either rodent predators or ant secondary dispersers [25]. However, we still have little experimental evidence showing how the outcomes of fruit handling by frugivores that vary in their effectiveness as dispersers affect secondary removal of diaspores and the potential escape-related benefits of dispersal [23].

Plant species in the Neotropical genus *Miconia* Ruiz & Pav. (Melastomataceae) produce typically ornithochoric berries and their seed dispersal and discard by avian frugivores have been extensively documented [26–30]. Ant species are frequently reported removing fruits of *Miconia* on the ground [31,32], as well their seeds from bird droppings [13,15], with substantial contribution as secondary seed dispersers in some cases [30,33]. In addition, rodents and marsupials also consume *Miconia* fruits, acting as predators and seed dispersers [34–36]. Spontaneous germination of *Miconia* seeds within fruits is unlikely to occur [37], thus seed release from fleshy pulp by birds [37,38], small mammals [34,36] and ants [31,32] is essential for successful germination. Therefore, *Miconia* comprise an ideal model to explore the consequences of variation in diaspore handling by birds for secondary removal by ground-dwelling fauna.

Here, we report a field study using the treelet *Miconia irwinii* Wurdack as a model to explore the effects of diaspore handling by avian frugivores on the short-term removal by the ground-dwelling fauna in *campo rupestre* vegetation, southeastern Brazil. We performed direct observations on fruit handling behavior of avian frugivores to determine their relative contribution to diaspore dispersal and discard beneath parent plants, quantified patterns of seed fall under treelets and recorded interactions between animals and diaspores on the ground. Additionally, we conducted factorial field experiments manipulating: (1) different outcomes of primary fruit/seed removal by birds, (2) distances of diaspore deposition from conspecifics, and (3) the access of ants and vertebrates to diaspores, in order to determine their independent and interactive effects on secondary diaspore removal. We hypothesized that the way avian frugivores discard and the place where they deposit seeds should affect short-term removal by ground-dwelling ants and vertebrates. More specifically, we expected that removal should be lower for gut-passed seeds within bird feces than for unpassed seeds directly removed from fruit pulp, because passage through the gut of dispersers is known to reduce seed attractiveness and detectability by potential seed predators [22,23]. Alternatively, gut-passed seeds could be also attractive for ant secondary dispersers, thus we could also expect higher removal of gut-passed than unpassed seeds [24,25]. Additionally, assuming that handled fruits with exposed fleshy pulps become more attractive to ground-dwelling animals [20,24], we expected that partially defleshed fruits simulating pulp pecking by birds should experience higher removal rates than intact fruits. We also expected that diaspores deposited near the parent plants should experience higher removal than those deposited far, assuming positive density-dependent effects of diaspore deposition near parent plants on attractiveness to ground-dwelling fauna [1]. Finally, considering the current reports of *Miconia* fruit and seed consumption by ground-dwelling vertebrates [34–36], we expected that diaspore removal should be higher when accessible to both vertebrates and ants than when accessible exclusively to ants.

## Materials and methods

Legal permission for ant and plant collection provided by ICMBio (Instituto Chico Mendes de Conservação da Biodiversidade). The legal permission for conducting feeding trials with captive birds housed in CETAS (Centro de Triagen de Animais Silvestres) provided by Instituto Estadual de Florestas de Minas Gerais (IEF-MG).

### Study site and species

We carried out the fieldwork in Vellozia Reserve, a private protected area in Serra do Cipó, Minas Gerais, south-eastern Brazil (43°35’W, 19°17’S, elevation 1150–1300 m a.s.l.). The study site comprises *campo rupestre* ecosystems (S1 Fig), old growth fire-prone tropical vegetation established on quartzite-derived rocks, with shallow, sandy and severely nutrient-impoverished soils, mostly in mountaintop areas above 900 m [39]. *Campo rupestre* harbors a diversified flora, including many endemic and threatened species, especially in the families Asteraceae, Poaceae, Orchidaceae, Melastomataceae and Fabaceae [39]. A vegetation mosaic alternating small riparian forests, grasslands and rocky outcrop patches characterize the landscape at the study site (S1 Fig). At Serra do Cipó the annual precipitation is around 1.300 mm and the annual average temperature is approximately 21°C [40]. The climate is Cwb according to Köppen-Geiger classification [41] characterized by a striking seasonality, with most of the rainfall occurring from October to April in the hot summers [40].

*Miconia irwinii* is a Brazilian endemic treelet found exclusively in rocky outcrops (S1 Fig) [42]. The ripe fruits are purplish-black berries measuring 7.6 ± 0.7 mm (mean ± SD, range 4.1–9.5 mm, N = 493) in diameter, the water content is approximately 68% of the fruit weight that is 0.206 ± 0.05 mg [32] and 0.032 ± 0.007 mg (N = 448) pulp dry weight. The number of seeds per fruit ranges from one to 14 (6.07 ± 2.7, N = 219) and seed dry weight is 0.004 ± 0.001 mg (range 0.001–0.008, N = 525). Crop size is variable but large individuals may produce up to 4,000 fruits (1,548 ± 1,307, mean crop size ± SD, N = 24) [43]. Fruit maturation occurs asynchronously within individual treelets, but the fruiting period is relatively synchronous among treelets within population, occurring from June to October and peaking in August/September during the cold dry season [40,43]. Primary seed dispersal of *M*. *irwinii* is carried out by bird species that vary in their effectiveness as seed dispersers [43]. Fruits falling beneath treelet canopy can be removed by ant species, which may transport seeds to their nests, clean seeds and discard them into refuse piles [32]. The seeds are nondormant [44] and do not germinate spontaneously when enclosed within the fruit pulp, thus seed depulping by birds [37] or ants [32] is necessary for germination.

### Fruit handling, diaspore discard and dispersal by birds

In order to record fruit handling behavior of avian frugivores, in May 2015 we randomly selected 15 reproductive *M*. *irwinii* treelets within a 25-ha plot, with plants separated by at least 60 m. In the fruiting peak from July to September 2015 we conducted 240 h of observation, 16 h for each treelet distributed in bouts of four hours of continuous observation, two bouts in the morning (07:00−11:00 AM) and two in the afternoon (1:00−5:00 PM). Each observation bout was performed by a single observer that recorded the feeding behavior of bird species using binoculars and stopwatches [43]. We recorded the identity of birds and counted the number of fruits handled during each visit classifying diaspores as swallowed, dropped under parent plants, or partially defleshed when birds remove just pieces of the fleshy pulp without ingesting seeds (S1 Fig).

We classified bird species according to their fruit handling behavior. We considered as gulpers those able to swallow entire fruits or mashers those that mash or peck fruits before swallowing pulp and seeds [6,43]. We expressed these results as the simple percentage of the total fruits handled by different disperser species that where swallowed, dropped or partially defleshed during frugivory observations. We also quantified patterns of seed dispersal from focal plants by visually estimating the minimum flight distance of birds’ post-feeding displacements [18], according to the following distance intervals: 0 m if any fruit was dropped or just pecked during the visit, > 0–30, > 30–50, > 50 m.

### Seed fall beneath fruiting treelets

In order to determine the amount of seeds falling under *M*. *irwinii*, we arbitrarily selected nine fruiting plants distant at least 30 m from each other to install traps to intercept falling diaspores. In each plant we installed five suspended traps attached to their fruiting shoots and to adjacent plants by twines coated with sticky barrier to avoid diaspore removal by invertebrates (S2 Fig). Traps consisted of filter paper cones attached to wire circles of 10 cm in diameter (area of 0.00785 m^2^), totaling a sampling area of 0.039 m^2^ under each plant canopy (S2 Fig). The area under the treelets with seed traps was estimated through the ellipse formula considering the two largest perpendicular projections of the canopy and the average area under treelet canopies was 1.03 ± 0.74 m^2^ (Mean ± SD, range 0.37–2.87, N = 9), with our seed traps covering approximately 4% of the area under fruiting trees. We monitored the diaspore traps weekly during 30 days in August/September in 2015 during the peak of fruit ripening [40,43].

We placed the material retrieved from traps in plastic bags, and then in the freezer, later we examined the material in the lab under the stereoscope. We classified seeds in the following categories: (i) pulp-free seeds—seeds completely separated from fleshy pulp; (ii) defleshed fruits—seeds within ripe fruits with pulp partially eaten by birds; (iii) intact fruits—seeds within undamaged ripe fruits; (iv) gut-passed seeds–seeds within bird droppings. We recorded only visually intact seeds and disregarded seeds within green unripe fruits or seeds with clear signs of pre-dispersal predation (empty seeds presenting perforations). We reported these results as the simple percentage of the total seeds falling on traps for each category. We also divided the total amount of seeds recorded by 30 (the number of sampling days) and reported the variance in number of seeds falling per m^2^ per day (seeds/m^2^/day) with treelets as sample unities (N = 9).

### Secondary diaspore removal experiments

During the fruiting peak in September 2016, we performed two field experiments to assess the effects of diaspore handling, deposition distance from conspecific and exclusion of vertebrates on secondary removal of seeds and fruits deposited on the ground.

The first experiment consisted in assessing the effect of gut passage (seed ingestion plus presence in bird feces) on seed removal. We first offered *M*. *irwinii* fruits to captivity birds and then collected their feces containing seeds (see S1 Text for detailed descriptions of seed collection and feeding trials with captivity birds). We then compared the removal of gut-passed seeds embedded in bird feces with removal of cleaned seeds manually extracted from fruit pulp (pulp-free seeds). In the second experiment, we removed part (20–25%) of fruit fleshy pulp using a plier, simulating fleshy pulp pecking by birds, and then compared the removal of these partially defleshed fruits with that of intact fruits. In order to avoid effects of human scent on diaspore removal [45], we always handled seeds and fruits using latex gloves during the experimental setup.

In order to assess the effect of dispersal distance from a conspecific in both experiments, we selected *M*. *irwinii* individuals distant at least 50 m from each other, and then we set 12 sampling blocks near (30–40 cm from the crown) to these plants and another 12 sampling blocks 25 m far, making sure that blocks were distant at least 25 m from any other fruiting conspecific. We chose this deposition distances because we found that most post-feeding displacement flights of avian dispersers occurred at distances within 30 m from the mother plants. We placed diaspore piles in sand tracking stations of 30 x 30 cm, made with sifted white sand collected from a nearby site (S3 Fig). We always placed the sand stations above horizontal portions of soil in crevices between rock blocks, avoiding placing stations in the top of rock blocks where they could be subject to displacements by winds. In order to allow visualization of tracks left by the ground-dwelling fauna and to avoid gravitational movements of the diaspores, we compacted and flattened each sand station horizontally before depositing diaspore piles and wire structures. All sand stations were set in the field at least 24h before placing seeds for the first experiment. We used the same sand stations in the fruit removal experiment in the subsequent day after finishing seed removal experiment, thus at least 24h after handling sand stations in the search for seeds.

To assess the contribution of ants and vertebrates we compared removal among diaspores accessible to ants and vertebrates (open treatment) and excluded from vertebrates but accessible to ants (caged treatment). We constructed wire exclosure cages (17 x 17 x 8 cm) fenced with wire mesh (1.2 cm) [18,30] and the wire cage structures without mesh to control for possible effects of wire presence on diaspore removal. In each tracking station, we placed one diaspore pile for each seed treatment (gut-passed vs. pulp-free seeds) or fruit treatment (partially defleshed vs. intact fruits), with two piles distant 10 cm apart from each other within tracking stations. We randomly selected one tracking station from each pair to place the fenced cage covering the diaspore piles. To control the possible effects of wire structure on diaspore removal, the neighboring tracking station also received a wire structure without fence covering diaspore piles (S3 Fig).

**Fig 1:**
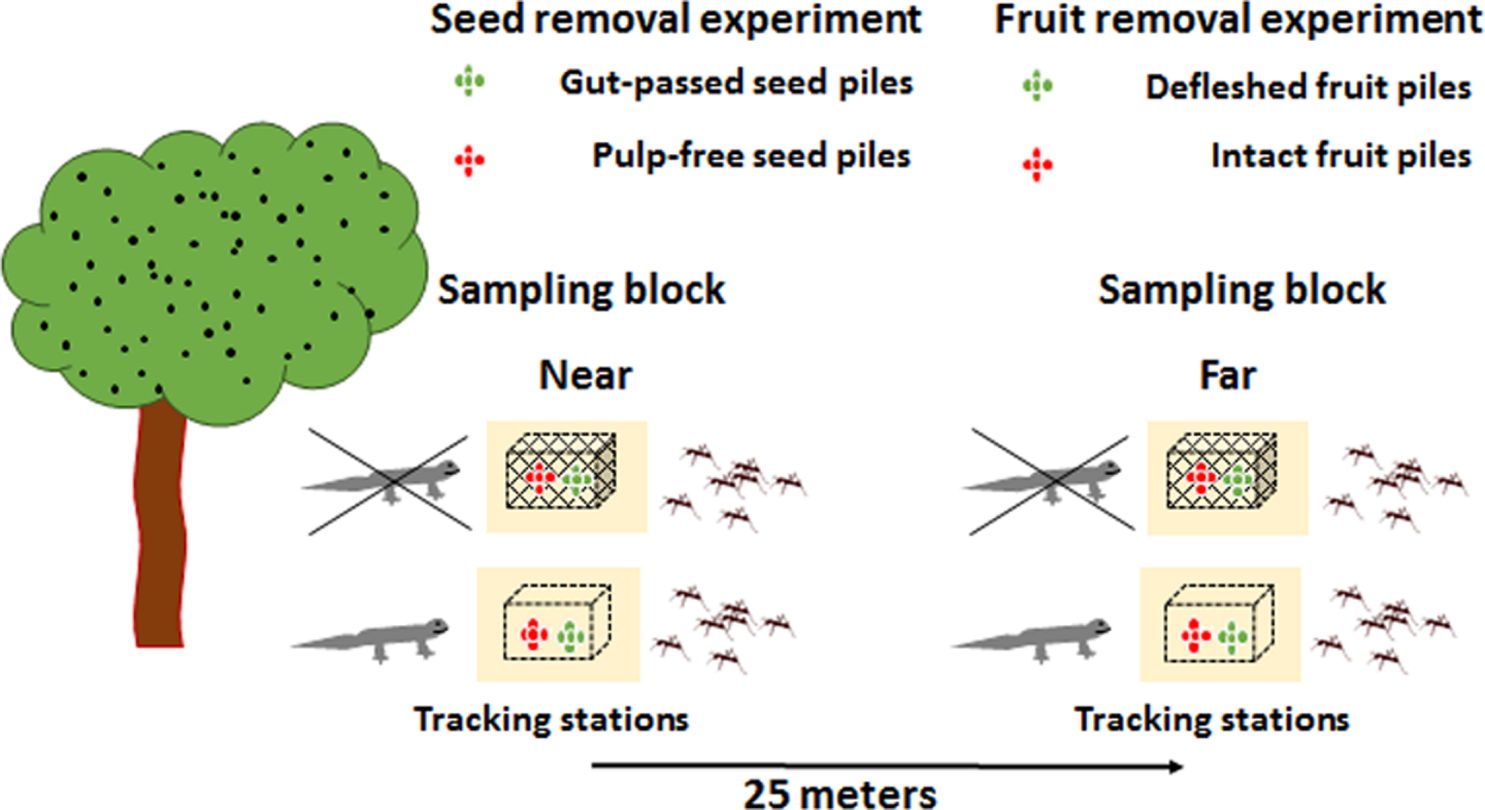
Schematic representation of the factorial experimental design employed to access the effects of diaspore handling, distance of deposition and vertebrate exclosure on removal of *Miconia irwinii* seeds and fruits by the ground-dwelling fauna in *campo rupestre* vegetation. Distribution of diaspore piles across eight treatment combinations, including two treatments simulating outcomes of diaspore handling by birds paired on each tracking station. We made diaspore piles in one tracking station accessible to ants plus vertebrates, whereas the nearby tracking station with diaspore piles were excluded from vertebrates but accessible to ants. We placed one sampling block near and the other 25 meters far from a fruiting plant.

Each sampling block comprised two tracking stations close to each other (40–60 cm), one with diaspore piles caged and the other left open (S3 Fig), one block near fruiting plant and one block distant per plant (Fig 1). We excluded the blocks from a single site in the seed removal experiment due to technical problems. Thus, our sample size was 11 seed piles for the eight possible treatment combinations in the first experiment, and 12 fruit piles for each treatment combination in the second experiment (Table 1). We placed 15 seeds per pile totaling 1320 seeds in the first experiment (660 gut-passed vs. 660 pulp-free, Table 1), and 10 fruits per pile totaling 960 fruits in the second experiment (480 partially defleshed vs 480 intact, Table 1). We recorded the total number of remaining diaspores after 48h of exposure in the field after exhaustively searching for seeds and fruits in the sand stations. We inspected for animal tracks and recorded interactions with potential seed dispersers or predators within 24h and 48h after setting diaspores on the stations.

**Table 1:** Number of diaspores per pile for each treatment combination (total number in parenthesis) in the seed and fruit removal experiments.

Distance of deposition	Near		Far	
Vertebrate access	Open	Excluded	Open	Excluded
Seed removal experiment	Sampling blocks (N = 11)		Sampling blocks (N = 11)	
**Gut-passed seeds**	15 (165)	15 (165)	15 (165)	15 (165)
**Pulp-free seeds**	15 (165)	15 (165)	15 (165)	15 (165)
Fruit removal experiment	Sampling blocks (N = 12)		Sampling blocks (N = 12)	
**Partially defleshed fruits**	10 (120)	10 (120)	10 (120)	10 (120)
**Intact fruits**	10 (120)	10 (120)	10 (120)	10 (120)

### Diaspore interactions with ground-dwelling ants

To assess interactions between ants and diaspores we performed direct observation bouts on diaspore piles placed in the ground. We observed diaspore piles close to 20 *M*. *irwinii* individuals distant at least 25 m from each other. We obtained and handled the diaspores used in observation bouts exactly as described in the diaspore removal field experiments. In each observation bout, we placed a pair of diaspore piles, one containing 15 seeds manually extracted from pulp and another pile of 15 gut-passed seeds embedded in bird feces. We repeated exactly the same procedures with partially defleshed and intact fruits, but with piles containing five fruits each. Each bout comprised one hour of continuous observation, totaling 40 h of observation, 20 h for paired seed piles (gut-passed vs. pulp-free seeds) and 20 h for paired fruit piles (partially defleshed vs. intact fruits). We performed observation bouts during the daytime, always between 10:00 AM and 05:00 PM. Each observation bout was performed by a single observer that recorded the time elapsed between placing diaspores on the ground and discovery by ants (latency time) in each treatment, the identity of ant species interacting with seed piles and their behavior handling diaspores. We followed the ants that effectively removed diaspores from piles and recorded dispersal distances whenever possible. We collected ants in glass vials with alcohol 70% and then prepared the material for identification in the laboratory under the stereoscope. To identify ant species, we compared the material to a reference collection from long-term research in this study site [46].

### Statistical analyses

We employed generalized linear mixed effects models (GLMMs, *glmer* for non-normal datasets, with *lme4* package in R) with fixed and random effects to analyze the datasets of seed and fruit removal experiments [47]. In each model, diaspore handling, deposition distance from conspecific, vertebrate exclosure and possible interactions among these factors were considered as the fixed effects. Sampling blocks were nested within plant and grouped as random effects to account for the spatial heterogeneity of samples [48]. The response variables were the proportions of seeds or fruits removed from diaspore piles after 48h of exposure in the field, for each experiment. We performed analyses assuming binomial distribution error of the response variable.

We built minimal models accounting for variation in dependent variables by employing backward exclusion of non-significant variables and interactions and through comparisons with null models, also performing post-hoc comparisons among treatments [47]. We used Student paired *t*-tests to compare latency time of ant-diaspore interactions and the number of interacting ant species between pulp-free and gut-passed seeds, and between partially defleshed and intact fruits. We performed all statistical analyses in R [49].

## Results

### Fruit handling, diaspore discard and dispersal by birds

**Fig 2:**
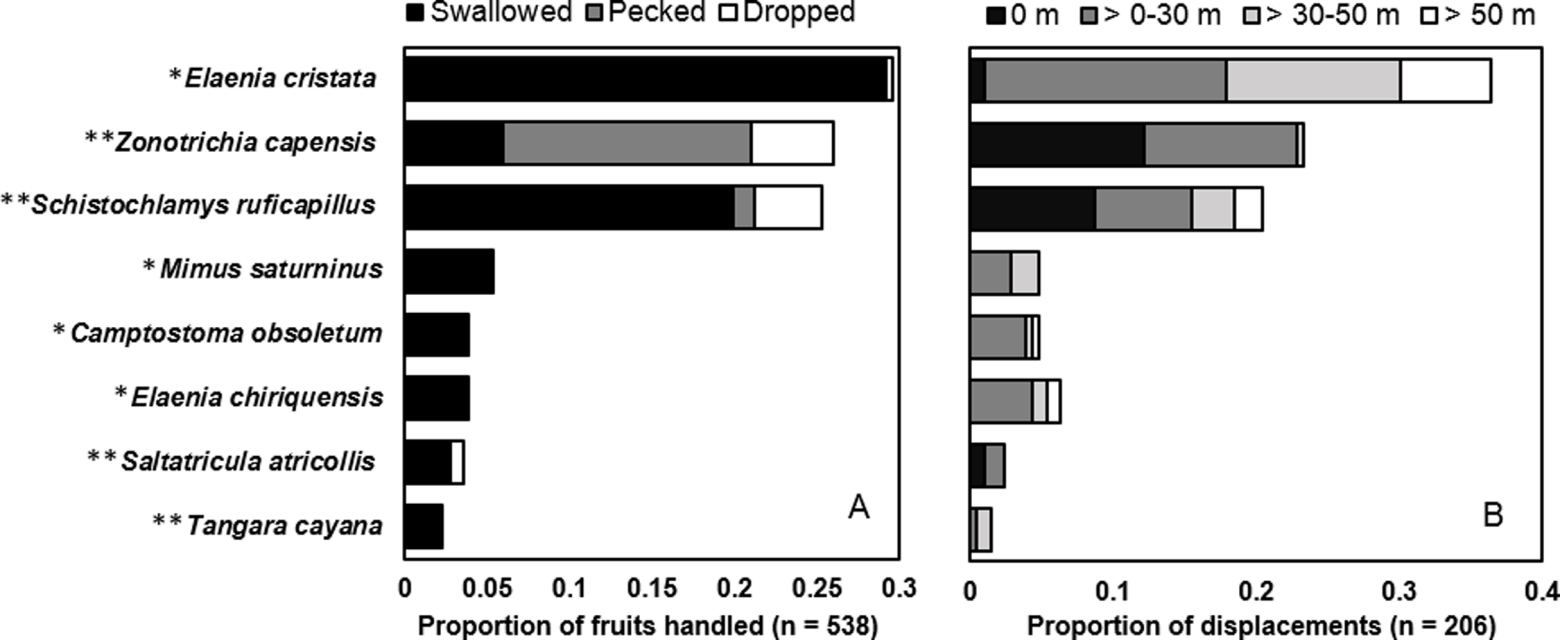
Handling, discard and dispersal of *Miconia irwinii* diaspores by avian frugivores in *campo rupestre* vegetation. (A) Relative contribution of bird species to seed dispersal through endozoochory (fruit ingestion) or diaspore discard near parent plants (fruit pecking or drop). (B) Estimated flight distance of post-feeding displacements made by potential seed dispersers. *Elaenia obscura* is a gulper, but it was not represented in the figure because it contributed to less than 1% of fruit removal. * Gulpers, ** Mashers.

We recorded nine bird species feeding on *M*. *irwinii* fruits (Fig 2), including five gulpers: *Elaenia cristata*, *Elaenia chiriquensis*, *Elaenia obscura*, *Camptostoma obsoletum* (Tyrannidae) and *Mimus saturninus* (Mimidae); and four mashers: *Zonotrichia capensis* (Passerellidae), *Schistochlamys ruficapillus*, *Saltatricula atricollis* and *Tangara cayana* (Thraupidae). Gulpers usually ingested the entire fruits and likely discarded the seeds embedded in their feces. Mashers were also able to ingest fruits by handling and squeezing fruit fleshy pulp before ingestion, and some seeds were ingested within fruit pulp and discarded through defecation. However, fruit mandibulation by mashers also resulted in seed ejection from fruit flesh, and pulp-free seeds either got stuck to the bill of the mashers or were dropped on the ground beneath treelet (S4 Fig). Two mashers, *S*. *ruficapillus* and *Z*. *capensis*, also pecked part of the fleshy pulp without detaching fruits from the plant, but frequently dropped the pecked fruits under the parent plants. Mashers accounted for 57% of the fruits handled, while gulpers accounted for 43% (n = 538). Gulpers swallowed nearly 42% and dropped less than 1% of the total fruits handled, while mashers swallowed nearly 31%, pecked fleshy pulp 16% and dropped under treelets nearly 10% of the total fruits handled (Fig 2A). Birds dropped at least one pecked fruit in approximately 23% of feeding events recorded, particularly two frequent masher species *S*. *ruficapillus* and *Z*. *capensis* (Fig 2A). Post-feeding displacements made by avian frugivores indicated that nearly 47% of seeds were dispersed at least within 30 m from parent plants (Fig 2B).

### Seed fall beneath fruiting treelets

**Fig 3:**
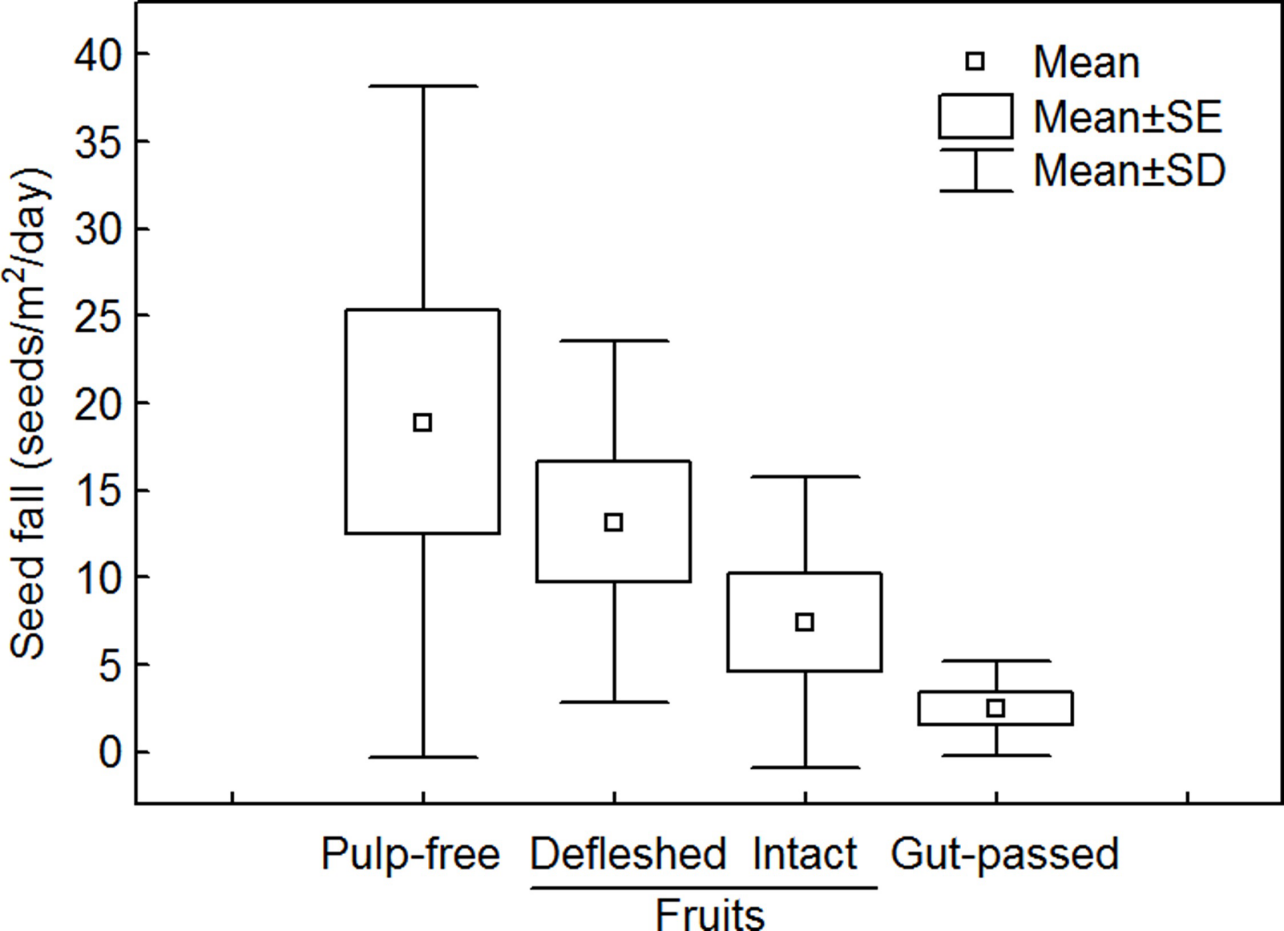
Variation in number of seeds falling under fruiting *Miconia irwinii* treelets in *campo rupestre* vegetation.

We estimated an average of 41.9 ± 9.4 seeds/m^2^/day (mean ± SE, range 9.4–110.2) falling under fruiting treelets. Among these, nearly 45% were pulp-free seeds, 32% in partially defleshed fruits, 17% inside intact fruits and only 6% embedded in bird feces (Fig 3).

### Secondary diaspore removal experiments

**Fig 4:**
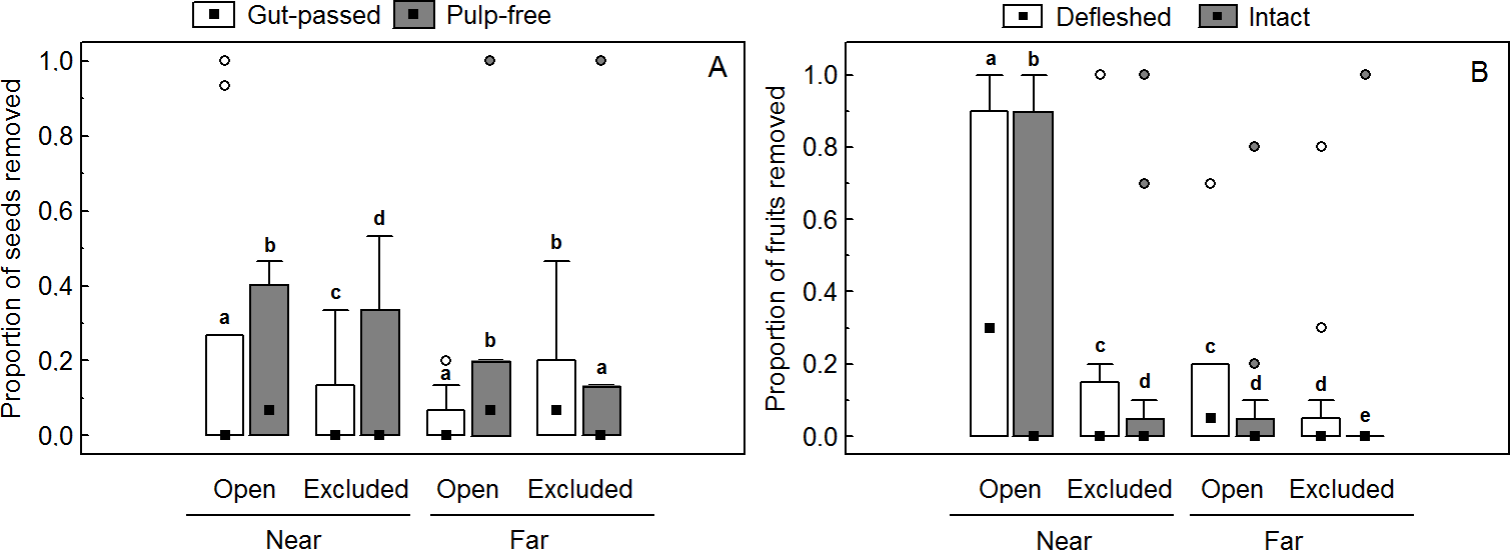
*Miconia irwinii* diaspore removal by the ground-dwelling fauna in *campo rupestre* vegetation. (A) Effects of gut passage (gut-passed vs. pulp-free seeds), distance from conspecific (near vs. far) and vertebrate exclosure (open vs. excluded) on the proportion of *Miconia irwinii* seeds removed. (B) Effects of fruit handling (partially defleshed vs. intact fruits), distance from conspecific and vertebrate exclosure on the proportion of *Miconia irwinii* fruits removed. Squares represent medians, boxes represent 25–75% percentiles, whiskers represent non-outlier ranges and circles show the outliers. Distinct letters denote significant differences among treatments.

In the seed removal experiment, only 172 (13%) of the 1320 seeds were removed after 48h. We found significant effects of gut passage and vertebrate exclosure on seed removal and a significant interaction between these factors, with no clear effect of diaspore deposition distances (Table 2 and Fig 4A). The removal of pulp-free seeds was higher (15%) than removal of gut-passed seeds (11%). Similarly, the removal of seeds accessible to vertebrates and ants was higher (15%) than removal of seeds excluded from vertebrates (11%). The interaction between gut passage and vertebrate exclosure was statistically significant (Table 2). Pulp-free seeds were more removed (17.5%) than gut-passed seeds (12.4%) when accessible to ants and vertebrates. However, we found lower differences between removal of pulp-free (12%) and gut-passed seeds (10%) when excluded from vertebrates. In two tracking stations with missing pulp-free seeds, we found evidence of the presence of the Brazilian guinea pig *Cavia aperea* (Caviidae, Rodentia), including its footprints and feces (S5 Fig).

**Table 2:** Results of generalized linear mixed effects model (GLMM). The effects of gut passage (GT), deposition distance from conspecifics (DS), vertebrate exclosure (VE) and their interactions (:) among these variables on seed removal. Also, the effects of fruit handling (FH), deposition distance from conspecifics (DS), vertebrate exclosure (VE) and their interactions (:) on fruit removal. Significance estimated by comparing the minimal model (MM) with the null model (NM). We built the minimum models through backward exclusion of non-significant variables (p > 0.05). The Akaike’s information criterion (AIC) represents the uncertainty of the model whereby lower AIC values represent the more parsimonious models. Error distribution used was binomial.

Response variables	Explanatory variables of the minimal model	D.f.	AIC (MM)	AIC (NM)	P
Seed removal	GT + VE + GT:VE	6	407.84	413.29	< 0.001
Fruit removal	FH + DS + VE + DS:VE	7	219.94	311.31	< 0.001

In the fruit removal experiment, 19% of the 960 fruits were removed after 48h. We found significant effects of fruit handling, dispersal and vertebrate exclosure on fruit removal, as well a significant interaction between distance of deposition and vertebrate exclosure (Table 2 and Fig 4B). Partially defleshed fruits were more removed (22%) than intact fruit (16%). Fruit removal near fruiting plants was higher (27%) than distant (11%), and higher when accessible to ants and vertebrates (25%) than when vertebrates were excluded (13%). Partially defleshed fruits deposited near fruiting plants and accessible to ants and vertebrates were more removed (39%) than intact fruits (29%). We found a significant interaction between distance from conspecific and vertebrate exclosure, with fruit removal near treelets being much higher for fruits accessible to ants and vertebrates (37%) than in the vertebrate exclosure treatment (17%). However, differences in fruit removal between fruits accessible to ants and vertebrates (13%) and excluded from vertebrates (9%) treatments were less marked for fruits deposited far from conspecifics.

During the experiment, we recorded a Blattodea species and the ant *Ectatomma edentatum* biting pieces of the fruit pulp, but we did not record diaspore removal. However, we found tracks of lizards on five stations accessible to vertebrates with missing fruits (S5 Fig). Lizards were frequent in the study site and we recorded three species near fruit piles, including *Tropidurus montanus*, *Eurolophosaurus nanuzae* (Tropiduridae) and *Ameivula cipoensis* (Teiidae) (S5 Fig). We noticed the presence of *M*. *irwinii* seeds in feces of *T*. *montanus*. In addition, in one station with missing fruits we also recorded many bird footprints (S5 Fig).

### Diaspore interactions with ground-dwelling ants

**Fig 5:**
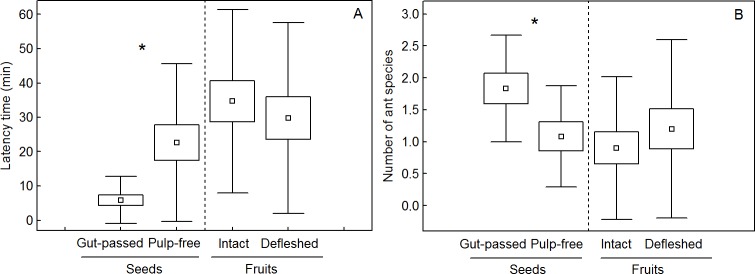
Ants interacting with *Miconia irwinii* diaspores on the ground of *campo rupestre* vegetation. (A) Latency time (time elapsed between placing diaspores on the ground and the first interaction with ants) across different experimental treatments. (B) Number of ant species interacting with diaspore piles. Squares represent mean, boxes standard error and whiskers standard deviation), asterisk denotes significant differences (Paired *t* test—p < 0.001).

We found 16 ant species in four subfamilies interacting with diaspores of *M*. *irwinii* on the ground, 11 species attended to seed piles and 12 species attended to fruit piles (Table 2). *Camponotus rufipes* and *Cephalotes pusillus* were the most frequent species and interacted with all diaspore treatments (Table 3 and S5 Fig). We recorded removal of gut-passed seed piles by *C*. *rufipes* in four observation bouts (S1 Film), and removal by *C*. *pusillus* and *Camponotus westermanni* in one occasion each (Table 3). These ant species examined mostly the fecal material and did not remove pulp-free seeds, but they carried bird droppings containing seeds up to 3 m (Table 3). We recorded the removal of pulp-free seeds from piles by *Sericomyrmex* sp. (N = 1), *Cyphomyrmex rimosus* (N = 3) and *Atta* sp. (N = 1). These ants seemed to use seeds as food resources thus probably acting as seed predators, and they may transport seeds up to 2 m. Ants found gut-passed seeds four times faster than pulp-free seeds (*t* test = 3.7, df = 19, p < 0.0001, Fig 5A). We recorded nine ant species interacting with gut-passed and eight species interacting with pulp-free seeds (Table 3). The average number of ant species attending diaspore piles was higher for gut-passed than for pulp-free seeds (*t* test t = 4.7, df = 11, p < 0.0001, Fig 5B). We recorded eight ant species interacting with intact fruits and 12 species interacting with partially defleshed fruits (Table 3), but we did not record any fruit removal attempt during observations. *Camponotus rufipes* and *C*. *pusillus* were also the most frequent visitors, but they did not remove fruits or seeds from fruits. The latency time of diaspore discovery by ants did not differ among partially defleshed and intact fruits (*t* test = 1.01, df = 19, p = 0.32, Fig 5A) and the difference between the number of ant species attending to piles of partially defleshed and intact fruits was low and marginally non-significant (*t* test = 2.04, df = 19, p < 0.055, Fig 5B).

**Table 3:** Ant species interacting with *Miconia irwinii* diaspores on the *campo rupestre* ground. Values represent the proportion of diaspore piles that each ant species was observed (N = 20).

Species/Subfamilies	Seeds		Fruits		Removal distance
	Pulp-free	Gut-passed	Intact	Defleshed	
**Dolichoderinae**					
*Dorymyrmex goeldii*			0.05	0.05	
*Dorymyrmex cf*. *pyramicus*	0.05				
**Ectatomminae**					
*Ectatomma edentatum*	0.05	0.05	0.05	0.05	
**Formicinae**					
*Brachymyrmex pictus*				0.05	
*Camponotus cf*. *punctulatus*			0.05	0.05	
*Camponotus renggeri*		0.10		0.05	
*Camponotus rufipes*	0.35	0.85	0.45	0.50	83 cm (2–297)*^†^
*Camponotus vittatus*		0.05			
*Camponotus westermanni*	0.05	0.10		0.05	70 cm *
**Myrmicinae**					
*Atta* sp.	0.05				> 200 cm**
*Cephalotes pusillus*	0.45	0.70	0.10	0.20	40 cm *
*Cyphomyrmex rimosus*	0.05	0.15	0.10	0.10	> 100 cm**
*Mycocepurus goeldii*			0.05	0.05	
*Pheidole radoszkowskii*	0.05	0.10	0.10	0.15	
*Sericomyrmex* sp.		0.05			> 80 cm**
*Wasmannia auropunctata*				0.05	

* Removal of gut-passed seeds,

** Removal of pulp-free seeds,

† mean/range (N = 4).

## Discussion

Our study highlights that variation in fruit handling by primary avian seed dispersers mediate subsequent interactions among discarded diaspores and ground-dwelling animals, potentially affecting final seed fates. Tyrant flycatchers and the chalk-browed mockingbird are gulpers that act as legitimate seed dispersers of *M*. *irwinii*, ingesting whole fruits and defecating most seeds away from parent plants. Tanagers and the rufous-collared sparrow are mashers and seem to play dual roles in this seed dispersal system. They can ingest whole fruits and effectively disperse seeds within their feces, but they also discard diaspores under parent plants by squeezing out the seeds during fruit handling or by dropping partially consumed fruits containing seeds. In fact, these patterns of fruit handling by birds have been previously documented for other *Miconia* species [28,30,38]. Moreover, we showed that most diaspores falling under parent plants comprise cleaned seeds ejected from the pulp or seeds inside the partially defleshed fruits. Gut-passed seeds defecated by effective dispersers are likely to interact with ant species that behave mostly as secondary dispersers, whereas pulp-free seeds were removed mostly by fungus-growing ants and rodents, regarded as potential seed predators. Therefore, we found that when birds ingest and defecate seeds they might benefit plants by maximizing predator escape and secondary dispersal. Conversely, birds that drop the partially defleshed fruits can increase probability of secondary removal by ants and vertebrates, which may act as rescuers of these discarded diaspores. Therefore, the removal of partially defleshed fruits by ant and vertebrate rescuers, including lizards and birds, could reduce the costs of interactions with less effective dispersers that drop the fruits under parent plants.

We hypothesized that the removal of discarded diaspores would be higher near parent plants, but our results challenged a simplistic interpretation of removal rates to unravel escape-related benefits in seed dispersal systems [1]. For instance, we found no clear differences in removal of gut-passed or pulp-free seeds deposited near or far from parent plants. However, our data partially corroborate the predictions of the Janzen-Connell model, because pulp-free seeds usually discarded by mashers beneath parent plants are likely to be removed by potential seed predators. Conversely, gut-passed seeds discarded distant from the parent plants were more removed by ant secondary dispersers. Additionally, we found higher removal of fruits deposited near parent plants than fruits deposited in farther areas. Nevertheless, this result indicates that distance-dependent effects on interaction frequency predicted by Janzen-Connell model [1] does not lead exclusively to higher seed predation or pathogen attack near parent plants, but it might also lead to higher removal of discarded fruits by potential secondary seed dispersers. Therefore, escape-related benefits of dispersal seems to be contingent on how primary dispersers handle and discard seeds.

We found that secondary removal of diaspores was highly variable at population scale, with relatively low removal rates by the ground-dwelling fauna (13% of seeds and 19% of fruits) when compared to other studies evaluating short-term removal of *Miconia* diaspores on ground [30,32]. These might relate to some caveats of our methodology, which might not reflect natural removal rates of diaspores discarded by frugivores. First, our estimate of removal rates after 48h may have underestimated real removal rates because discarded diaspores can be available for consumption by the ground-dwelling fauna for much longer periods and substantial removal could also occur within one or two weeks [21–23]. Moreover, our experimental setup involved small-scale disturbances due the use of sand tracking stations. This could have affected diaspore detectability by chemically- or visually-oriented animals. However, it is important to note that possible effects of tracking station disturbance on diaspore detectability and removal were spread in all samples, allowing us to evaluate the differences in removal rates among treatments.

We found that seed passage through the gut of avian frugivores reduced secondary removal rates. We also found that seed removal was significantly higher when accessible to both ants and vertebrates than when accessible exclusively to ants. Thus, vertebrates significantly contributed to seed removal, particularly the seed predator *C*. *aperea*. Moreover, we found that removal of pulp-free seeds was higher than that of gut-passed seeds, and this pattern was more evident with diaspores accessible to ants and vertebrates then when we excluded vertebrates. These results indicate that mammal seed predators seem to avoid gut-passed seeds. Our data are in line with evidence provided by previous studies highlighting that benefit of diaspore treatment in the guts of frugivores is also related to changes in seed condition that might reduce seed removal by potential predators [22,23,25]. However, the mechanisms behind this pattern are still underappreciated. We need further studies scrutinizing the effects of changes of seed condition due to the removal of chemical attractants during gut passage [23] and the effects of presence of feces mediating the attraction or repellence of predators and secondary seed dispersers.

Vertebrate exclusion indicated that ants were responsible for most removal of the pulp-free and gut-passed seeds. We observed that seeds embedded in bird feces were found more quickly and attracted more ant species than pulp-free seeds, in agreement with previous studies [24]. However, we showed that being more attractive does not necessarily implies higher removal rates. Indeed, gut-passed seeds were less removed than pulp-free seeds, even when accessible exclusively to ants. These counterintuitive results could be explained by the identity and behavior of ants removing gut-passed and pulp-free seeds. From the 11 ant species observed interacting with seeds, including *Pheidole*, a common predator of *Miconia* seeds [15,16,30], only six removed seeds. Pulp-free seeds were removed only by *Cyphomyrmex rimosus*, *Sericomyrmex* sp and *Atta* sp., species that supposedly use seeds to grow their fungus inside the nests [50–52]. Some leaf-cutting ants may act as secondary dispersers of bird-dispersed plants, because they use the fruit pulp to grow their fungus and discard viable seeds in refuse piles where seedling recruitment is more likely to occur [32–33]. Since we observed these ants removing pulp-free seeds, it is unlikely that they will place the seeds in refuse piles, thus they probably use seeds as the resource and act as predators [13].

Gut-passed seeds were removed by ant species (*C*. *rufipes*, C. *westermanni*, *C*. *pusillus*) that did not remove pulp-free seeds. In fact, it seems that fecal material elicits recruitment of these ants that could be attracted mostly to the nitrogenous wastes or undigested material present in bird feces [53]. Two frequent ant species acting as secondary seed dispersers, *C*. *rufipes* and *C*. *pusillus*, are known to play a central role in ant-plant interaction networks in *campo rupestre* vegetation [54]. Those ants are not granivorous and feed mostly on liquid resources, mainly extra-floral nectar and insect honeydew [54,55]. Remarkably, *C*. *rufipes* usually monopolized the piles of gut-passed seed and behaving aggressively upon other ant species (S1 Film). Besides acting as an important secondary disperser, this species could also negatively affect the removal of gut-passed seeds by granivorous ants. Therefore, our results corroborate studies showing that handling behavior of avian frugivores directly affect the condition of the discarded seeds, which might alter the identity of ant species interacting with diaspores deposited on the ground, possibly affecting seed fate [22,23,25].

We found that removal of fruits deposited near conspecifics was much higher than in far locations. Fruit fall is unlikely to occur far from parent plants and this result points out that the ground-dwelling fauna is able to track resource availability on resource rich patches. We found that mashers drop considerable amounts of pecked fruits under parent plants and that partially defleshed fruits were significantly more removed than intact fruits. Consumption of fleshy pulp by frugivores that do not remove fruits from plants failing to disperse the seeds may affect negatively subsequent interaction between plants and other primary seed dispersers [56]. Conversely, our results indicate that less effective primary seed dispersers might have positive effects on subsequent interactions among discarded diaspores and the ground-dwelling fauna.

Vertebrate exclusion significantly reduced fruit removal, highlighting that this group might contribute up to 55% of the fruits removed near fruiting conspecifics. When accessible to both ants and vertebrates, partially defleshed fruits deposited near fruiting plants were significantly more removed than intact fruits. However, when accessible exclusively to ants the differences in removal of partially defleshed and intact fruits were non-significant. Ants found the partially defleshed and intact fruits in similar rates and we found no differences in average number of ant species attracted to fruit piles from both treatments, unlike previously reported for *Pyschotria* fruits Atlantic forest [24]. Therefore, it seems that fleshy pulp pecking by mashers makes discarded fruits more attractive to vertebrates than to ants.

Contrary to our expectations, we found no clear evidence of fruit removal by small mammals. Nevertheless, the common presence of lizards and their tracks on sand stations indicate that they could consume fruits discarded by primary dispersers near parent plants. In addition, we found visually viable seeds of *M*. *irwinii* in feces of *T*. *montanus*, indicating that these lizards could act as rescuers of diaspores deposited beneath the parent plants. *Tropidurus* lizards can be important seed dispersers in rocky ecosystems [57,58]. Although lizards are mostly insectivores, they ingest small amounts of fruits [59], including *Miconia* [60], especially during the dry season peak, which coincides with the fruiting period of *M*. *irwinii* [40]. Due to their high abundances in *campo rupestre* vegetation [59,61], we believe that the contribution of lizard species as secondary dispersers of *M*. *irwinii* seeds needs deeper investigation. Besides, we also recorded bird footprints indicating that some ground-dwelling species (e.g. Red-winged Tinamou, Spotted Nothura) could be involved in consumption of the fruits deposited near the parent plants. However, to understand the role played by lizards and birds as rescuers or predators of diaspores discarded by primary dispersers, we need to assess gut passage effects on seed germination, to track seed deposition and their potential contribution to seedling establishment [3].

Vertebrate exclusion indicate that the contribution of ants to fruit removal on the ground is also considerable, up to 100% in few cases. However, our results differs from other studies that reported most secondary removal of *Miconia* fallen fruits by ants [30,31]. For instance, a previous study accessing removal of *M*. *irwinii* intact fruits beneath parent plants found that ants were the main gatherers of *M*. *irwinii* fruits in *campo rupestre* ground [32]. The authors found only four ant species interacting with fruits, including records of removal by *C*. *rufipes* and *Atta sexdens*, with the later dispersing diaspores up to 46 m and depositing *M*. *irwinii* seeds in refuse piles outside their nests [32]. Conversely, we did not record any event of diaspore transport by ants, probably because our observations efforts were in the daytime and we missed activity of nocturnal species. The consequences of ant foraging activity on fallen fruits, which include secondary dispersal, seed cleaning and deposition on refuse piles, are known to be important for population dynamics of some bird dispersed plants [14,18,19]. Our study indicates we still need more detailed studies to determine the outcome of ant-fruit interactions and the actual contribution of ants as secondary seed dispersers or as predators of *M*. *irwinii*.

In conclusion, our study illustrates how complementary approaches are necessary to unravel the complexity behind two-phased seed dispersal processes [8,18]. Assessment of bird feeding behavior and the use of seed traps under parent plants allowed us to set plausible contexts regarding the outcomes of diaspore handling by avian primary seed dispersers. By combining observational and experimental approaches, we were able to infer what components of the ground-dwelling fauna potentially interact with diaspores handled by avian frugivores promoting their secondary removal. Continuous activity of ants and vertebrates upon gut-passed seeds dispersed away, pulp-free seeds or fruits with pecked fleshy pulp discarded near parent plants through the fruiting season could represent an important factor driving the recruitment dynamics of *M*. *irwinii* in *campo rupestre* vegetation. Lastly, our study calls for the attention of possible shifts in seed fate related to the effects of diaspore handling by avian frugivores that vary in their effectiveness as primary dispersers.

## Supporting information

S1 Fig Overview of the study site and plant species. A, B—Study site encompassing *campo rupestre* vegetation; C—*Miconia irwinii* treelet; D—Ripe fruits; E, F—Fruits with fleshy pulp partially eaten by birds.
(PDF)

S2 Fig Traps installed to intercept diaspores falling beneath the crown of *Miconia irwinii*. A, B, C—Fruiting individuals of *M*. *irwinii* with diaspore traps; D—Detail of a diaspore trap made with filter paper attached to wire circles; E—A twine coated with sticky barrier in detail; F—The Chalk-browed Mockingbird (*Mimus saturninus*) feeding on *M*. *irwinii* fruit above diaspore traps.
(PDF)

S3 Fig Experimental setup for assessment of diaspore removal in the field. A, B—Sampling blocks comprising two tracking stations close to each other, one with diaspore piles accessible to ants and vertebrates (open) and the other accessible exclusively to ants (caged); Wire exclosure cages with seed (C) and fruit piles (D); E—Wire cage structure without mesh for controlling possible effects of wire presence on diaspore removal; F—Simulation of fleshy pulp pecking by birds with the aid of a plier (Note the use of latex gloves to avoid human scent effects on diaspore removal).
(PDF)

S4 Fig Birds acting as primary seed dispersers of *Miconia irwinii* at Serra do Cipó, Brazil. The Cinnamon Tanager (*Schistochlamys ruficapillus*) (A) and the Black-throated Saltator (*Saltatricula atricollis*) (B) feeding on *M*. *irwinii* fruits, pulp-free seeds stuck to the birds’ bills in detail; the Rufous-collared Sparrow (*Zonotrichia capensis*) (C), the Plain-crested Elaenia (*Elaenia cristata*) (D) and the Lesser Elaenia (*Elaenia chiriquensis*) (E); F—Focal observation procedures to record avian frugivores, the blue arrow shows the observer and the green arrow the focal plant.
(PDF)

S5 Fig Ground-dwelling fauna recorded during diaspore removal experiments. A—The Brazilian Guinea Pig (*Cavia aperea*), its feces and footprints found on a tracking station with missing pulp-free seeds (B); The lizards *Tropidurus montanus* (C), *Eurolophosaurus nanuzae* (D) and *Ameivula cipoensis* (E) were frequently recorded near tracking stations; F—The ant *Camponotus rufipes* interacting with gut-passed seeds; G—Removal of pulp-free seed by the ant *Sericomyrmex* sp.; H—Bird footprints on tracking station with missing fruits.
(PDF)

S1 Text Supplementary methods. (PDF)

S1 Film The ant *Camponotus rufipes* as a secondary seed disperser of *Miconia irwinii*. Edited by AJA.
(MP4)

S1 Worksheet Data from “Handling by avian frugivores affects diaspore secondary removal”. (XLSX)
