# Supplementary material for: Handling by avian frugivores affects diaspore secondary removal
Source: PLoS One. 2018 Aug 29;13(8):e0202435. doi: 10.1371/journal.pone.0202435 (PMC6114891; doi:10.1371/journal.pone.0202435)
Supplement: S1 Fig — A, B—Study site encompassing campo rupestre vegetation; C—Miconia irwinii treelet; D—Ripe fruits; E, F—Fruits with fleshy pulp partially eaten by birds. (PDF) [file pone.0202435.s001.pdf]

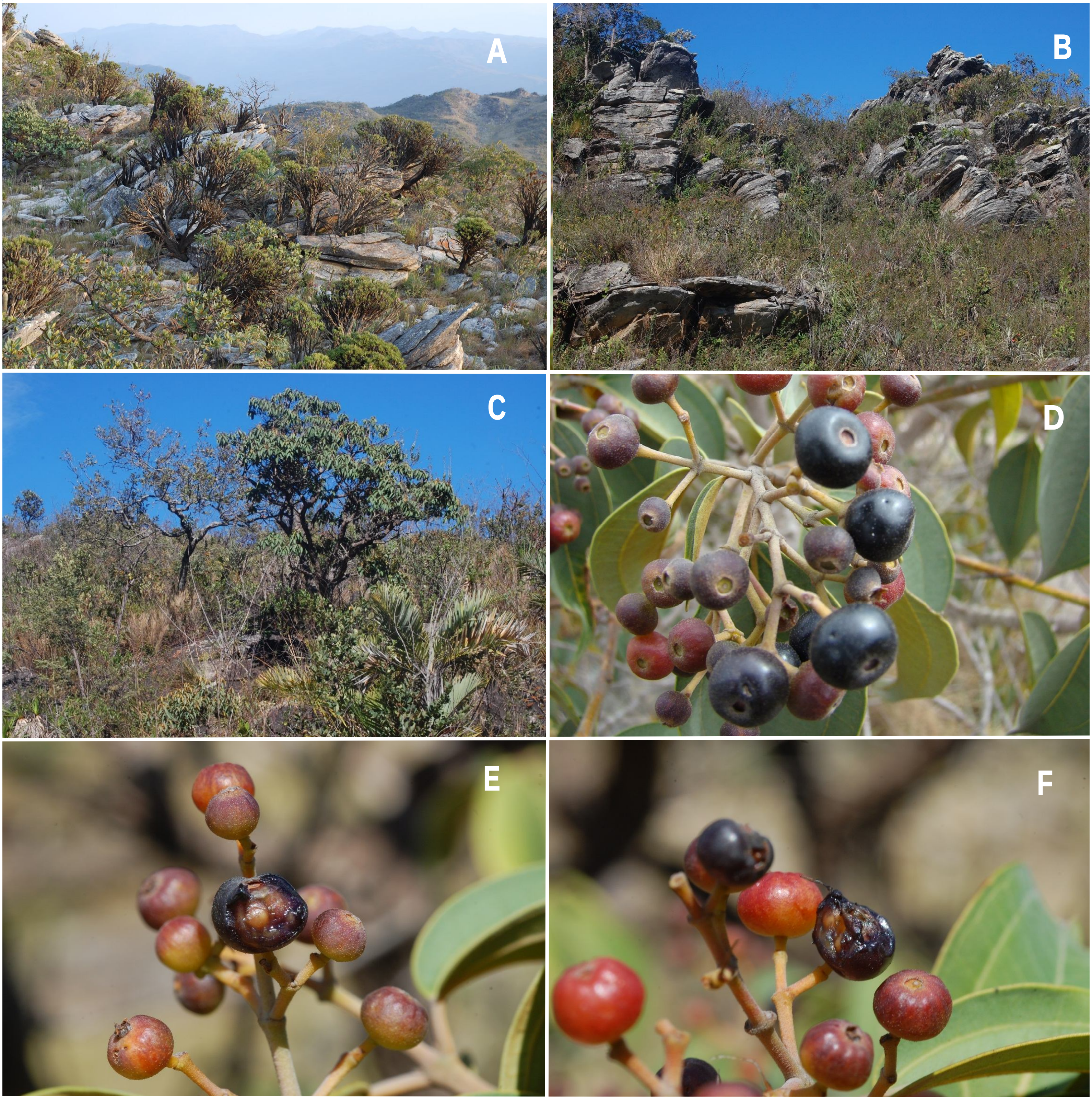

S1 Figure. Overview of the study site and plant species. A, B - Study site encompassing *campo rupestre* vegetation; C - *Miconia irwinii* treelet; D - Ripe fruits; E, F – Fruits with fleshy pulp partially eaten by birds.
