# Supplementary material for: Handling by avian frugivores affects diaspore secondary removal
Source: PLoS One. 2018 Aug 29;13(8):e0202435. doi: 10.1371/journal.pone.0202435 (PMC6114891; doi:10.1371/journal.pone.0202435)
Supplement: S2 Fig — A, B, C—Fruiting individuals of M. irwinii with diaspore traps; D—Detail of a diaspore trap made with filter paper attached to wire circles; E—A twine coated with sticky barrier in detail; F—The Chalk-browed Mockingbird (Mimus saturninus) feeding on M. irwinii fruit above diaspore traps. (PDF) [file pone.0202435.s002.pdf]

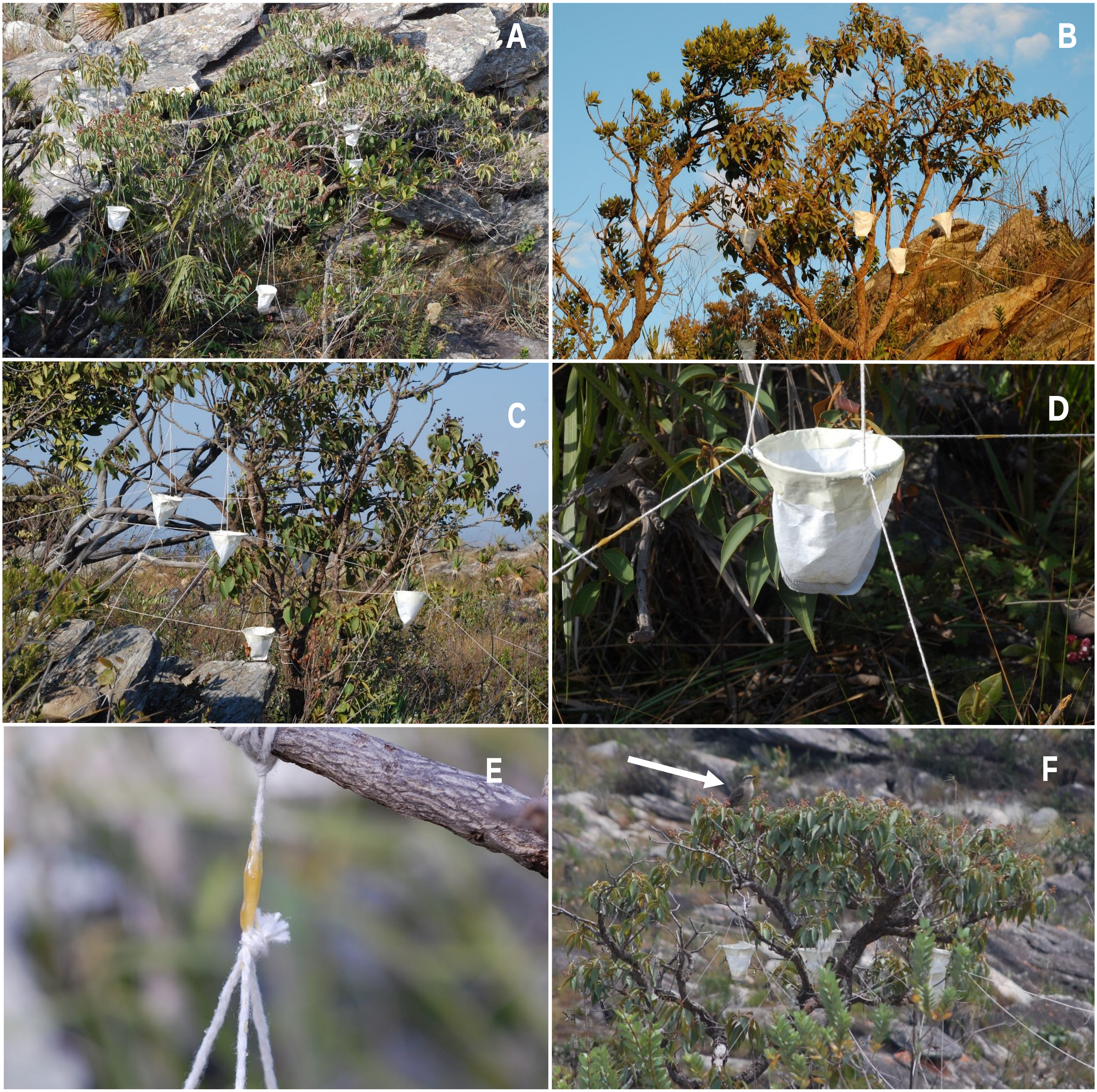

S2 Figure. Traps installed to intercept diaspores falling beneath the crown of *Miconia irwinii*. A, B, C - Fruiting individuals of *M. irwinii* with diaspore traps; D - Detail of a diaspore trap made with filter paper attached to wire circles; E - A twine coated with sticky barrier in detail; F - The Chalk-browed Mockingbird (*Mimus saturninus*) feeding on *M. irwinii* fruit above diaspore traps.
