# Supplementary material for: Handling by avian frugivores affects diaspore secondary removal
Source: PLoS One. 2018 Aug 29;13(8):e0202435. doi: 10.1371/journal.pone.0202435 (PMC6114891; doi:10.1371/journal.pone.0202435)
Supplement: S3 Fig — A, B—Sampling blocks comprising two tracking stations close to each other, one with diaspore piles accessible to ants and vertebrates (open) and the other accessible exclusively to ants (caged); Wire exclosure cages with seed (C) and fruit piles (D); E—Wire cage structure without mesh for controlling possible effects of wire presence on diaspore removal; F—Simulation of fleshy pulp pecking by birds with the aid of a plier (Note the use of latex gloves to avoid human scent effects on diaspore removal). (PDF) [file pone.0202435.s003.pdf]

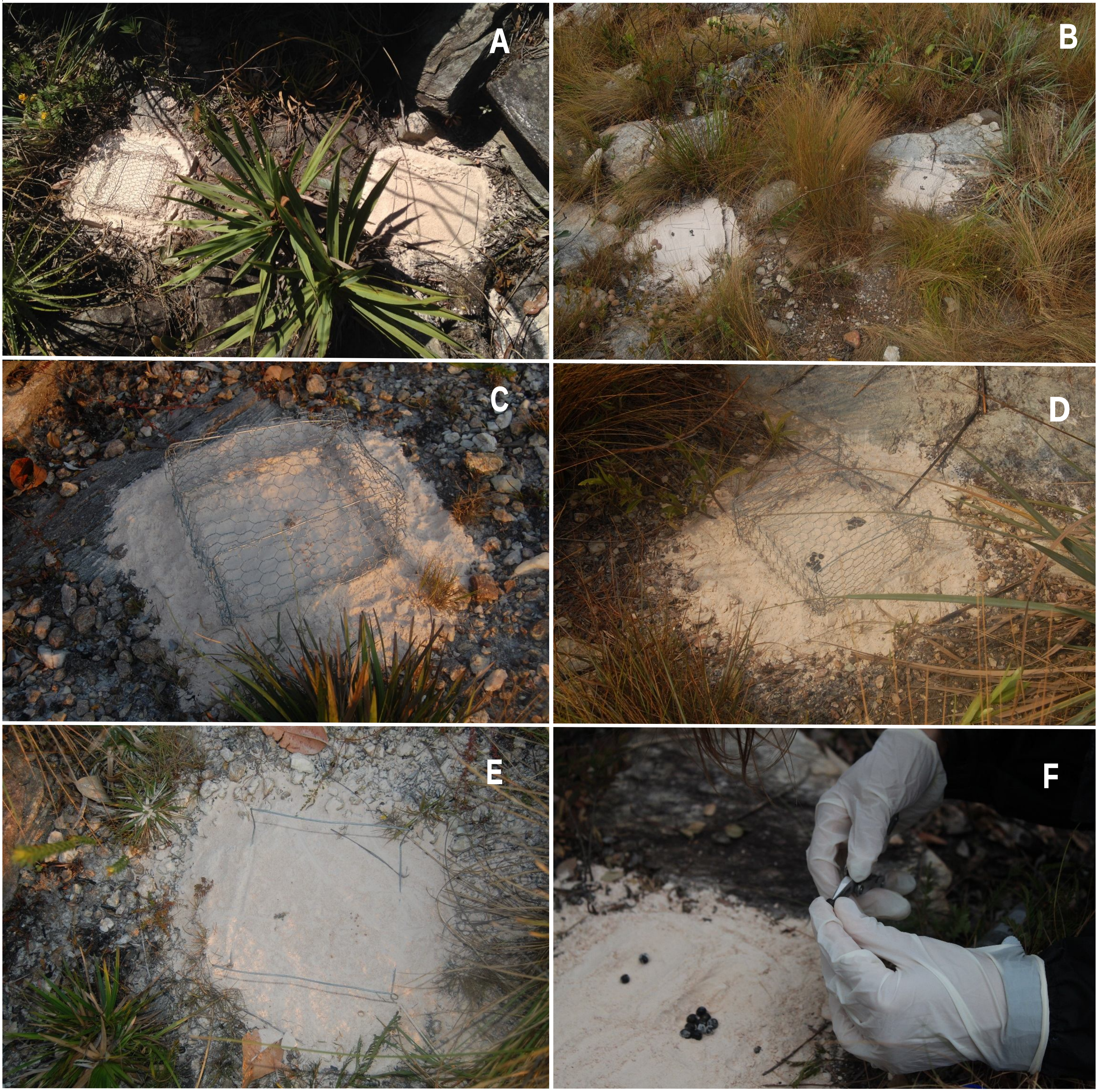

S3 Figure. Experimental setup for assessment of diaspore removal in the field. A, B - Sampling blocks comprising two tracking stations close to each other, one with diaspore piles accessible to ants and vertebrates (open) and the other accessible exclusively to ants (caged); Wire exclosure cages with seed (C) and fruit piles (D); E - Wire cage structure without mesh for controlling possible effects of wire presence on diaspore removal; F - Simulation of fleshy pulp pecking by birds with the aid of a plier (Note the use of latex gloves to avoid human scent effects on diaspore removal).
