# Supplementary material for: Handling by avian frugivores affects diaspore secondary removal
Source: PLoS One. 2018 Aug 29;13(8):e0202435. doi: 10.1371/journal.pone.0202435 (PMC6114891; doi:10.1371/journal.pone.0202435)
Supplement: S4 Fig — The Cinnamon Tanager (Schistochlamys ruficapillus) (A) and the Black-throated Saltator (Saltatricula atricollis) (B) feeding on M. irwinii fruits, pulp-free seeds stuck to the birds’ bills in detail; the Rufous-collared Sparrow (Zonotrichia capensis) (C), the Plain-crested Elaenia (Elaenia cristata) (D) and the Lesser Elaenia (Elaenia chiriquensis) (E); F—Focal observation procedures to record avian frugivores, the blue arrow shows the observer and the green arrow the focal plant. (PDF) [file pone.0202435.s004.pdf]

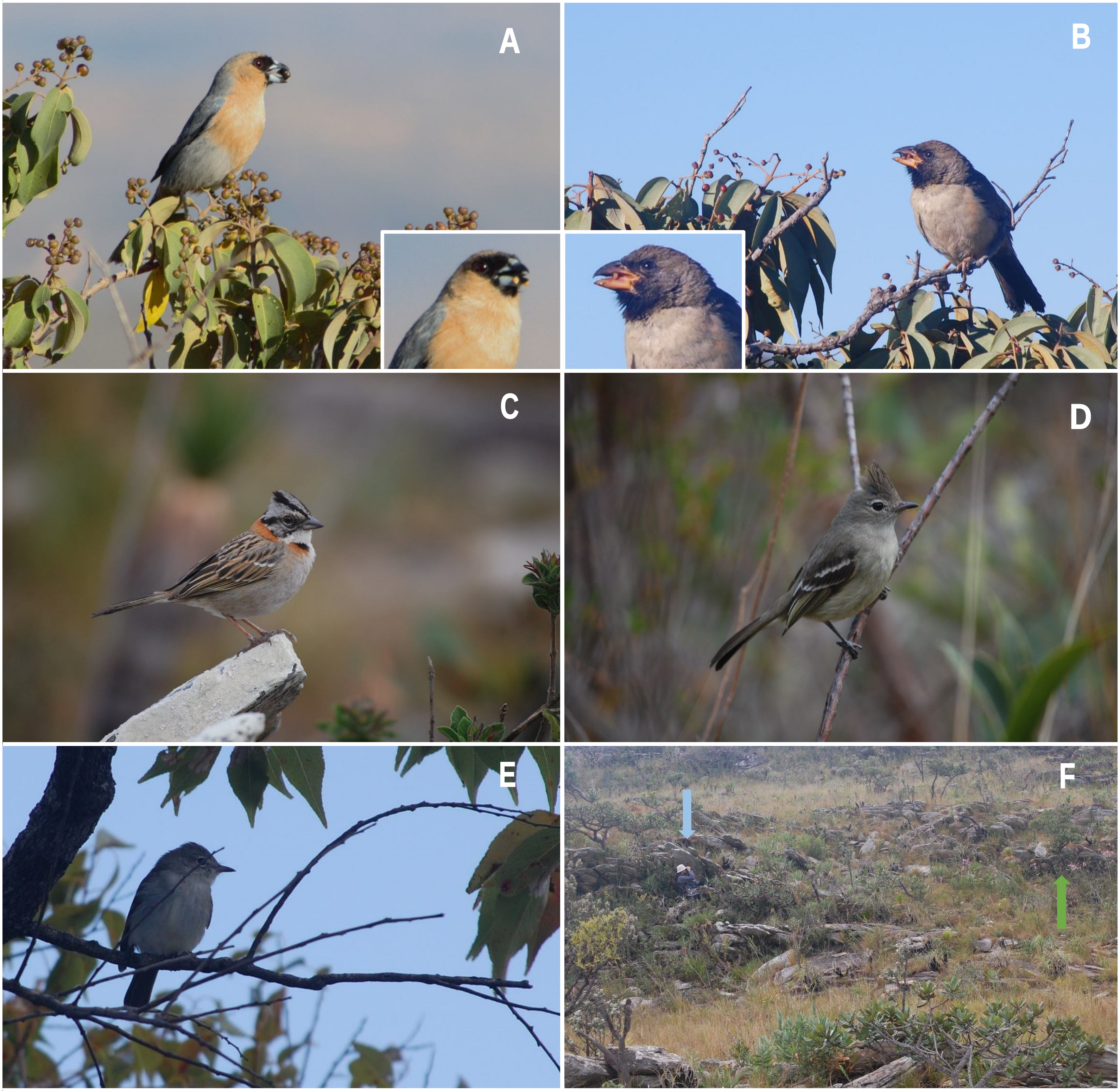

S4 Figure. Birds acting as primary seed dispersers of *Miconia irwinii* at Serra do Cipó, Brazil. The Cinnamon Tanager (*Schistochlamys ruficapillus*) (A) and the Black-throated Saltator (*Saltatricula atricollis*) (B) feeding on *M. irwinii* fruits, pulp-free seeds stuck to the birds' bills in detail; the Rufous-collared Sparrow (*Zonotrichia capensis*) (C), the Plain-crested Elaenia (*Elaenia cristata*) (D) and the Lesser Elaenia (*Elaenia chiriquensis*) (E); F - Focal observation procedures to record avian frugivores, the blue arrow shows the observer and the green arrow the focal plant.
