# Supplementary material for: Handling by avian frugivores affects diaspore secondary removal
Source: PLoS One. 2018 Aug 29;13(8):e0202435. doi: 10.1371/journal.pone.0202435 (PMC6114891; doi:10.1371/journal.pone.0202435)
Supplement: S5 Fig — A—The Brazilian Guinea Pig (Cavia aperea), its feces and footprints found on a tracking station with missing pulp-free seeds (B); The lizards Tropidurus montanus (C), Eurolophosaurus nanuzae (D) and Ameivula cipoensis (E) were frequently recorded near tracking stations; F—The ant Camponotus rufipes interacting with gut-passed seeds; G—Removal of pulp-free seed by the ant Sericomyrmex sp.; H—Bird footprints on tracking station with missing fruits. (PDF) [file pone.0202435.s005.pdf]

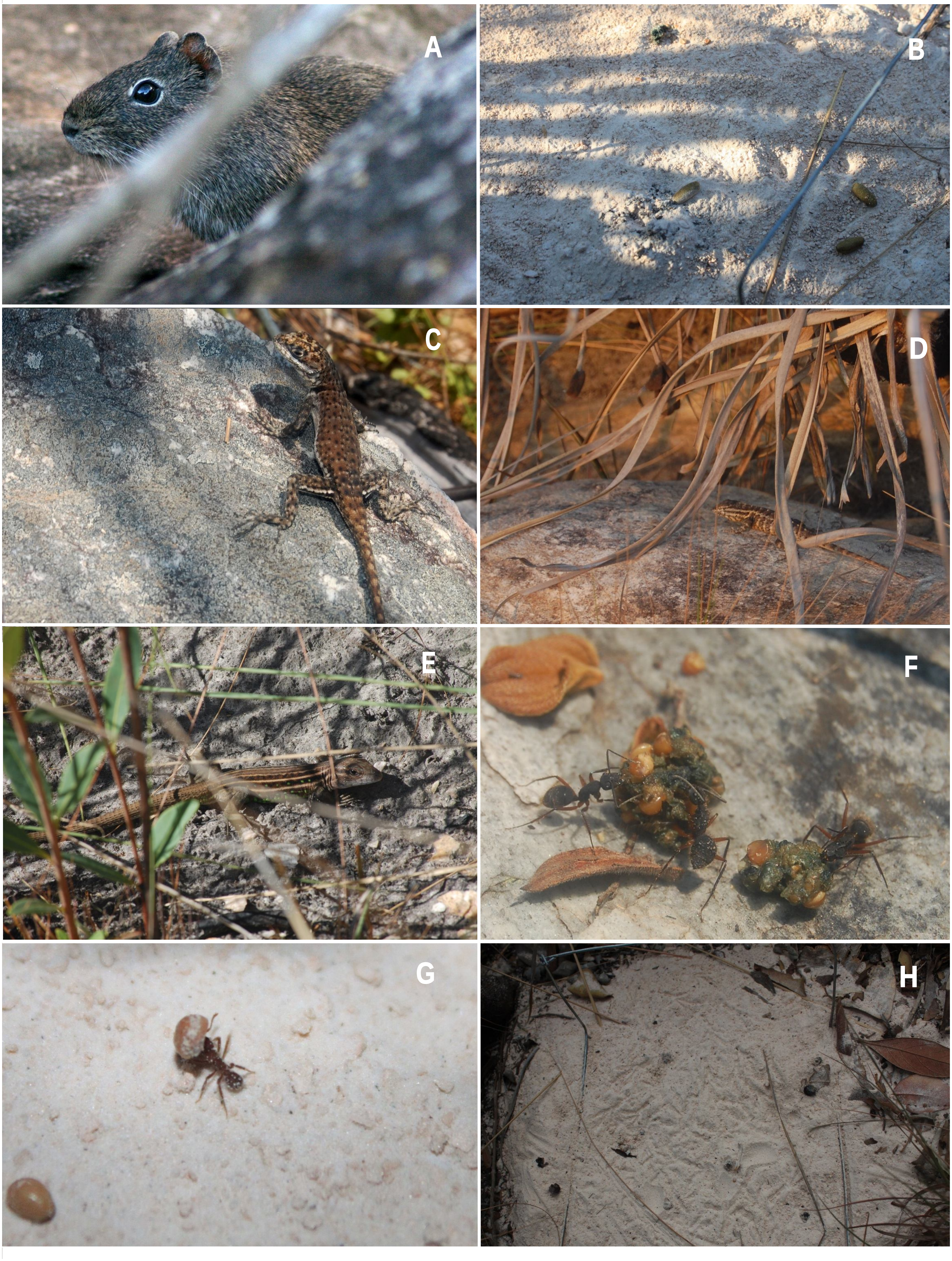
A
B
C
D
E
F
G
H

S5 Figure Ground-dwelling fauna recorded during diaspore removal experiments. A - The Brazilian Guinea Pig (*Cavia aperea*), its feces and footprints found on a tracking station with missing pulp-free seeds (B); The lizards *Tropidurus montanus* (C), *Eurolophosaurus nanuzae* (D) and *Ameivula cipoensis* (E) were frequently recorded near tracking stations; F - The ant *Camponotus rufipes* interacting with gut-passed seeds;
G - Removal of pulp-free seed by the ant *Sericomyrmex* sp.;
H - Bird footprints on tracking station with missing fruits.
