# Supplementary material for: Handling by avian frugivores affects diaspore secondary removal
Source: PLoS One. 2018 Aug 29;13(8):e0202435. doi: 10.1371/journal.pone.0202435 (PMC6114891; doi:10.1371/journal.pone.0202435)
Supplement: S1 Text — (PDF) [file pone.0202435.s006.pdf]

Supplementary methods

Feeding trials with captive birds and seed collection

In August 2016, we collected ripe fruits from five *M. irwinii* individuals at Serra do Cipó for subsequently feeding trails with captive passerine species (Fig 1) available in Centro de Triagem de Animais Silvestres (CETAS), a wildlife rehabilitation center from the Ministry of Environment of Brazil at the city of Belo Horizonte, Minas Gerais. For feeding trials, we followed similar procedures adopted in other studies that used captive birds from CETAS [1, 2]. We stored fruits in the freezer until the day before feeding trials with captive birds. We offered approximately 200 *M. irwinii* fruits to five bird species, including two individuals of *Thraupis episcopus* Linnaeus (Thraupidae), two individuals of *Thraupis sayaca* Linnaeus (Thraupidae), three individuals of *Turdus leucomelas* Vieillot (Turdidae), one individual of *Turdus rufiventris* Vieillot (Turdidae), and one individual of *Turdus amaurochalinus* Cabanis (Turdidae). We used birds already held in individual cages that were in good health conditions, from genera reported to feed on *Miconia* fruits. Before offering fruits to birds, we just covered cages' floor with filter papers (Fig 2) without handling birds. In captivity, the diet of these birds consisted mostly of banana and papaya, but during three consecutive days of feeding trails, we fed birds exclusively with *M. irwinii* fruits and the seeds embedded in their feces were collected (Fig 3). All birds consumed the offered fruits and defecated the seeds. We placed all samples within a single recipient (Fig 4) and used a plastic stick to homogenize the feces from different bird species, in order to avoid species-specific effects on seed removal. We divided the fecal mass obtained into portions of 15 seeds embedded in bird feces placing these samples in plastic wraps, which were stored in the freezer until the day of setting the

seed removal experiment in the field. We manually extracted the seeds from pulp, also separating them into 15 seeds portions in plastic wraps and stored at the same conditions for controlling the freezing effect. We handled seeds and fecal samples using latex gloves during experimental setup in order to avoid human scent effects on seed removal rates [3].

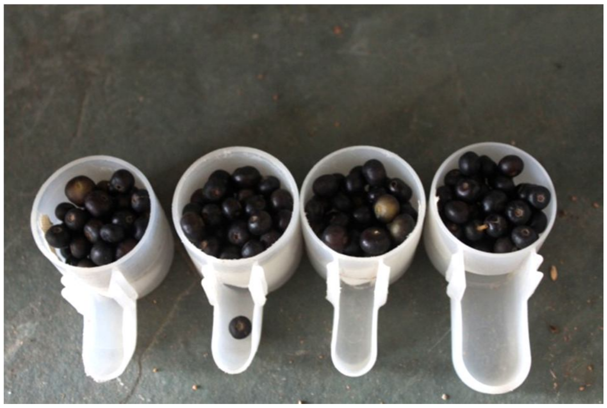

Figure 1. *Miconia irwinii* ripe fruits offered to captive birds.

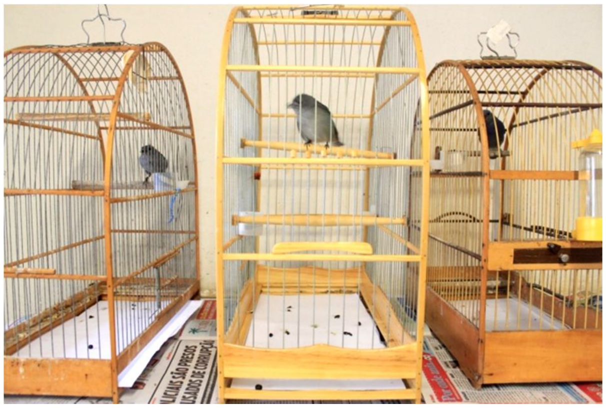

Figure 2. Captive birds fed on *M. irwinii* fruits.

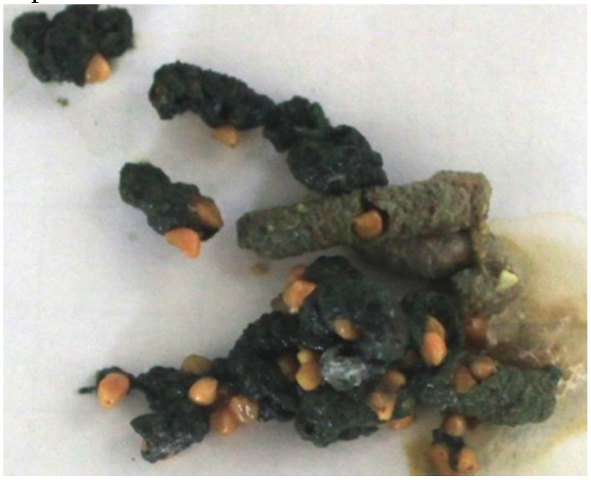

Figure 3. Feces of captive birds containing intact *M. irwinii* seeds.

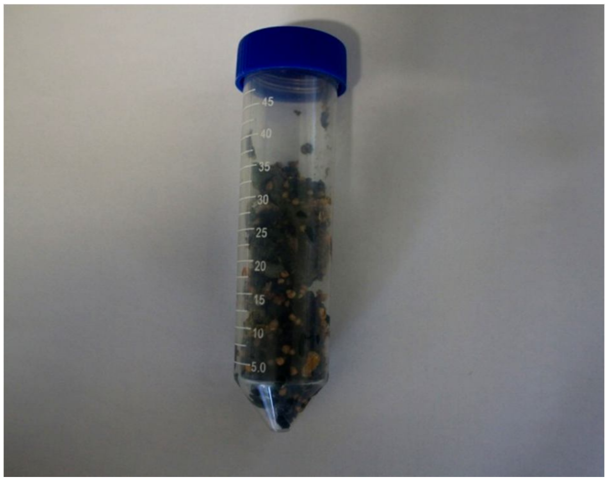

Figure 4. Homogenized fecal mass containing *M. irwinii* seeds passed through the gut of captive avian frugivores.

References

1- Silveira FAO, Mafia PO, Lemos-Filho JP, Fernandes GW. Species-specific outcomes of avian gut passage on germination of Melastomataceae seeds. Plant Ecol. Evol. 2017, 145: 350-355. https://doi.org/10.5091/plecevo.2012.706

2- Santos AMO, Jacobi CM, Silveira, FAO. Frugivory and seed dispersal effectiveness in two *Miconia* (Melastomataceae) species from ferruginous campo rupestre. Seed Sci. 2017. Res. https://doi.org/10.1017/S0960258517000071

3- Duncan RS, Wenny DG, Spritzer MD, Whelan CJ. Does human scent bias seed removal studies? Ecology. 2002; 83: 2630–2636
